# The Medical Profession and the Bill

**Published:** 1911-07-01

**Authors:** 


### The Medical Profession and the Bill.

Our leading article in The Hospital of June 10, in which we counselled the profession to study the details of the Government's Bill for Compulsory Sickness Insurance before condemning it off-hand, has evoked a few protests from readers who declare that they are entirely at one with the policy of those who oppose the measure tooth and nail. The case for and against the profession is being discussed so adequately by our contemporaries that we do not feel justified in throwing open our columns for further discussion on that aspect of the matter which more prominently concerns the doctor. The institutional aspect, on the other hand, is neglected elsewhere, and we think, therefore, that we are serving the cause of many by drawing attention to the vital points regarding which it is necessary to have further information.

We have no desire to water down or retract the statements which we made in the article to which some correspondents have taken objection. Nor do we think that we stand alone in voicing these statements. And, thirdly, we do not agree that they are open only to one interpretation—namely, that we have institutional interests more at heart than professional. The Insurance Bill is as weighty and important to the doctors as it is to the hospitals; it is going to modify very seriously the conditions under which both labour. Of that there can be no question. But we are the last in the world to advise a complete antagonism towards the Bill simply and solely because it is extremely likely that it will gravely change the present system of hospital relief. What we have urged upon hospital workers, and what we would urge upon our professional colleagues, is to study the Bill calmly and dispassionately, to pay no attention to the absurd and travestied statements that have been made regarding it, but to read its text for themselves and to annotate it freely. What we would warn both doctor and hospital worker against is to let the impression get abroad that if our demands for just and adequate treatment are not promptly met there is going to be a professional strike. Nothing can do greater damage to those legitimate interests which we are pleading for than an attitude of defiant unreasonableness such as is evidenced in the many letters which have been published in some quarters, and in which the most astonishing suggestions, from an organised petition against the Bill to the penalising by the General Medical Council of every practitioner who accepted the terms, have been revealed. There is not the least possibility that the Chancellor of the Exchequer will lower his wage-limit; on the contrary, there is every indication that his example of fixing it at £160 is going to be followed by Germany, where the limit is forty pounds less for invalidity insurance. The question of professional representation on the Commission we may safely leave to be thrashed out in Parliament. The demand that every insured person should have a free choice of doctor is one that has been specifically repudiated by the law in Germany and which it would be difficult to insist upon here. It can only be gained if the profession shows that its general demands are reasonable and made in the interests of the public as much as of itself. And at present, unfortunately, there is every indication that the public, as a whole, regards the doctors' opposition to the insurance scheme as an antagonism which is primarily founded on self-interest.

